# Ecology of leishmaniasis in the South of France. 22. Reliability and representativeness of 12 *Phlebotomus ariasi*, *P. perniciosus* and *Sergentomyia minuta* (Diptera: Psychodidae) sampling stations in Vallespir (eastern French Pyrenees region)

**DOI:** 10.1051/parasite/2013035

**Published:** 2013-10-11

**Authors:** Jean-Antoine Rioux, Stéphane Carron, Jacques Dereure, José Périères, Lamri Zeraia, Evelyne Franquet, Michel Babinot, Montserrat Gállego, Jorian Prudhomme

**Affiliations:** 1 Faculté de Médecine, Université Montpellier 1 1 rue École de médecine 34000 Montpellier France; 2 Entente Interdépartementale pour la Démoustication du Littoral Méditerranéen (EID-Méditerranée) 165 rue Paul Rimbaud 34000 Montpellier France; 3 Office National des Forêts (ONF) 505 rue de la Croix Verte, Parc Euromédecine 34094 Montpellier France; 4 Université Aix-Marseille, IMBE, Pôle de l’Étoile, Saint Jérôme 13397 Marseille Cedex 20 France; 5 Parasitology Laboratory, Faculty of Pharmacy Av. Joan XXIII 08028 Barcelona Spain; 6 UMR MIVEGEC (IRD 224 – CNRS 5290), Universités Montpellier 1 et 2 911 avenue Agropolis 34394 Montpellier France

**Keywords:** Leishmaniasis, Pyrénées-Orientales, Ecoepidemiology, Vector sampling, Phytoecological indicator, Climate change, Zero point

## Abstract

This study was conducted around Céret (Pyrénées-Orientales, mean elevation 200 m) to test the statistical reliability of 12 stations devoted to sampling the *Leishmania infantum* vectors *Phlebotomus ariasi* and *P. perniciosus* in the South of France. Each station included a retaining wall and the surrounding phytoecological environment (total area: 2,000 m^2^). The wall had rectangular drainage cavities (weep holes) in which flight interception traps (sticky paper) were inserted and stretched every 10 days from May to October. For both vector species, the statistical analysis of 10-day and annual frequencies led to the following conclusions: (1) *P. ariasi* densities were significantly higher than *P. perniciosus* densities, (2) densities per species were significantly different at the 12 stations : none of them could be considered as representative of local vector densities, which depend on the wall structure (exposure, shade, vertebrate hosts), (3) the 10-day variation trends were not significantly different between stations, indicating that these variations are not determined by the station structure but rather by a common external factor (likely meteorological) and (4) the phytoecological features at the stations were not correlated with the sandfly densities. Most of the observations obtained with *P. ariasi* and *P. perniciosu*s are also relevant for the non-vectorial species *S. minuta.* In conclusion, future research on the dynamics of leishmaniasis outbreaks relative to climate change and agricultural-silvicultural modifications should be very cautiously carried out, while focusing especially on the vector sampling quality and the use of phytoecological maps as vector density indicators.

## Abstract (fr)

La présente étude, réalisée aux environs de Céret (Pyrénées-Orientales, altitude moyenne 200 m) avait pour objectif d’éprouver la fiabilité de 12 stations de *Phlebotomus ariasi* et de *P. perniciosus,* vecteurs de *Leishmania infantum* dans le Sud de la France. Chaque station comportait un mur de soutènement et son environnement phyto-écologique (surface totale : 2000 m^2^). Le mur était pourvu de cavités rectangulaires dans lesquelles ont été insérés des pièges d’interception (papiers adhésifs) tendus tous les 10 jours, de mai à octobre. Pour les deux vecteurs étudiés, les analyses statistiques ont conduit aux déductions suivantes : 1) les densités de *P. ariasi* sont significativement supérieures à celle de *P. perniciosus,* 2) les densités de chaque espèce sont significativement différentes pour les 12 stations : aucune d’elles ne peut donc être considérée comme représentative des densités vectorielles locales; ces valeurs dépendent de la structure du mur (exposition, ombre portée, hôtes vertébrés), 3) le sens des fluctuations décadaires n’est pas significativement différent d’une station à l’autre : ces fluctuations ne sont donc pas conditionnées par la structure des stations, mais par un facteur commun extérieur, vraisemblablement de nature météorologique, 4) la phyto-écologie des stations n’est pas corrélée avec leur richesse en Phlébotomes. La plupart des observations obtenues avec *P. ariasi* et *P. perniciosus* sont également pertinentes pour l’espèce non-vectorielle *S. minuta*. En conclusion, les recherches sur la dynamique des foyers leishmaniens, en rapport avec les changements climatiques ou les modifications agro-sylvicoles, devraient être conduites avec beaucoup de prudence : une attention particulière devrait être accordée à l’échantillonnage des vecteurs et à l’utilisation des phytocénoses comme indicateurs des stations.

*« Il faut laisser l’expérience à sa liberté : c’est la tenir captive que de n’en montrer que le côté qui prouve et que de voiler le côté qui contredit ».*^1^

Denis Diderot

## Introduction

For several decades, the epidemiology of leishmaniasis has benefited from ecology-based scientific concepts and methods. The aim of this approach was to investigate epidemiological cycles in terms of “parasitic systems”. The ecoepidemiological method developed on this occasion [11, 23] enabled identification of key factors that govern the structure and function of leishmaniasis outbreaks. The disciplines involved in this approach include: 1° mesology (biotopes, biogeography, bioclimatology, predators and pathogens), 2° taxonomy, genetics and evolution, 3° trophic and sexual behaviours of sandflies (hematophagy, trophogonic cycle, “eurystenogamy”, fertility, fecundity and diapause). 4° morphophysiological modifications in parasites during the intravectorial cycle (multiplication, fusion, parietal attachment, metacyclogenesis), 5° *Leishmania* virulence in vertebrate reservoirs (receptivity, inoculation chancre, visceralization and immunity), 6° the last step of the approach, rational control, involves several techniques (physical, chemical and biological) targeting the cycle overall, i.e. the parasite, vectors and reservoirs.

This ecoepidemiological approach was applied in the Mediterranean region, which gave rise to the vector pre-eminence concept, i.e. the sandfly vector is the main factor responsible for the structure and dynamics of leishmaniasis outbreaks [23, 28].

**Figure 1.**
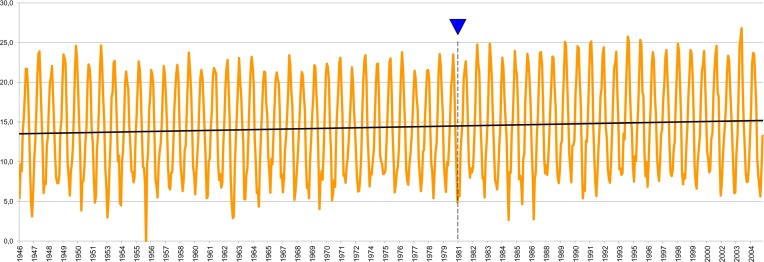
Variations in mean monthly temperatures at the Montpellier-Fréjorgues (France) meteorological station between 1946 and 2004 (blue triangle: 1981, year of the study). The data have a normal distribution and the regression is significant (trend curve: *Y* = 0.0024, *X* + 13.511). The temperature increase over the considered period was 1.895 ± 0.068 °C.

Global warming was recently taken into account with respect to the emergence and expansion of these outbreaks, thus giving new impetus to this type of research [5, 14, 16, 26]. However, to clearly confirm the involvement of climatic factors, project leaders have stressed the need to very precisely determine the conventional zero point. In Languedoc-Roussillon, where mean annual temperatures increased by around 2 °C between 1946 and 2004 (Figure 1), these recommendations prompted us to reassess the statistical quality of the trapping initiatives conducted in 1981 in the eastern Pyrenees region where both *Phlebotomus ariasi* and *P. perniciosus*, i.e. sandfly vectors of *Leishmania infantum*, are found. The aim of the present study was to detect potential sampling bias, which could call into question the conclusions of certain previous studies [22].

## Material and methods

### General points

**Figure 2.**
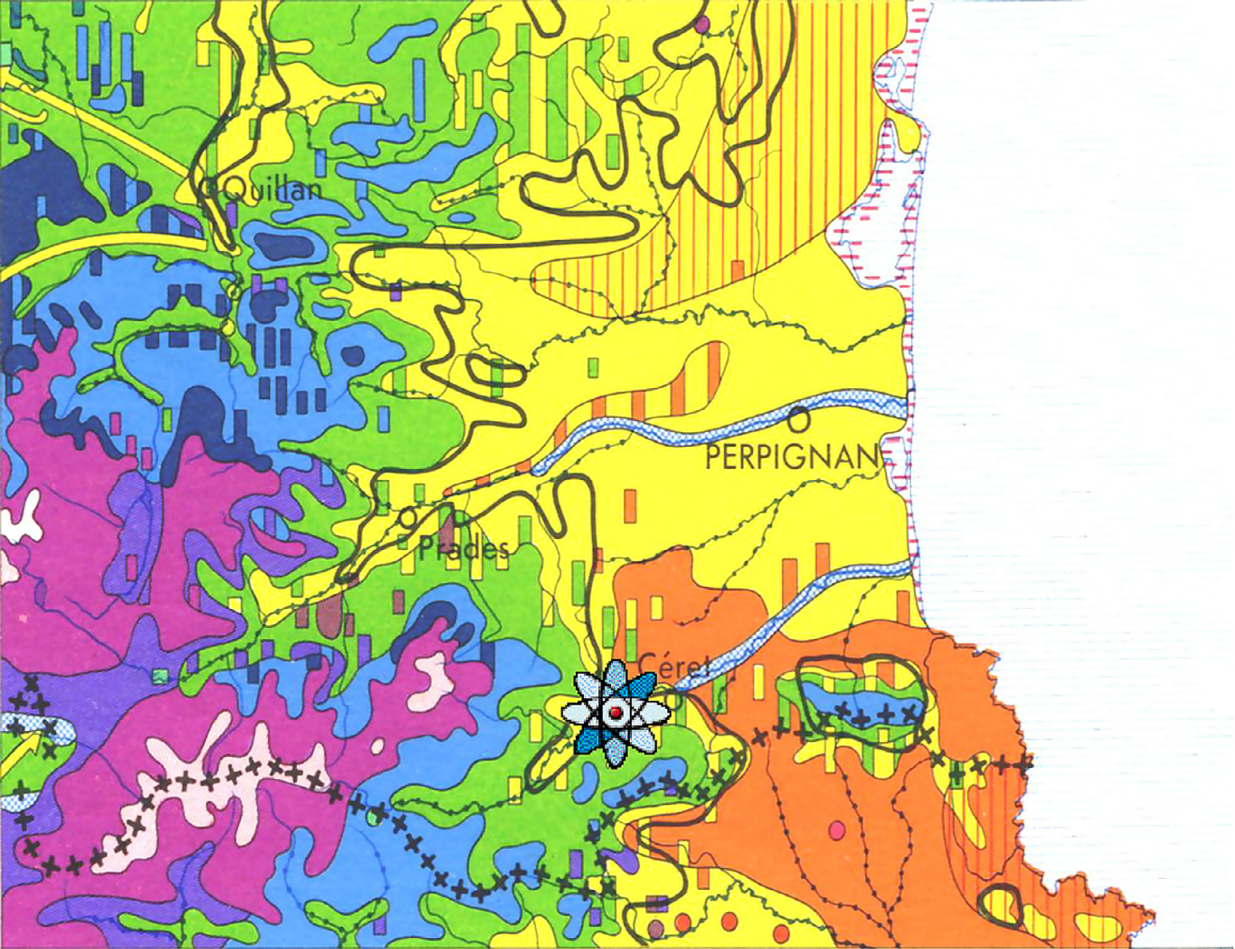
Map of botanical successions around Perpignan (*in*: H. Gaussen : Carte de la végétation de la France au 1 / 200 000^e^, CNRS). In orange: cork oak. In yellow: holm oak. In green: white oak. In light blue: beech. In dark blue: fir. In purple: Scots pine. In red: mountain pine. In pink: alpine storey. Sampling area, 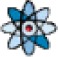, located in the Mediterranean holm oak and white oak succession. There were patches of cork oak.

The survey was carried out at Vallespir, in the vicinity of Céret (P.O., France), in an area of mixed oak, including *Quercus ilex*, *Q. pubescens* and *Q. suber* (Figure 2), which is typical subhumid Mediterranean climate [4, 8, 9, 13, 20].

**Figure 3.**
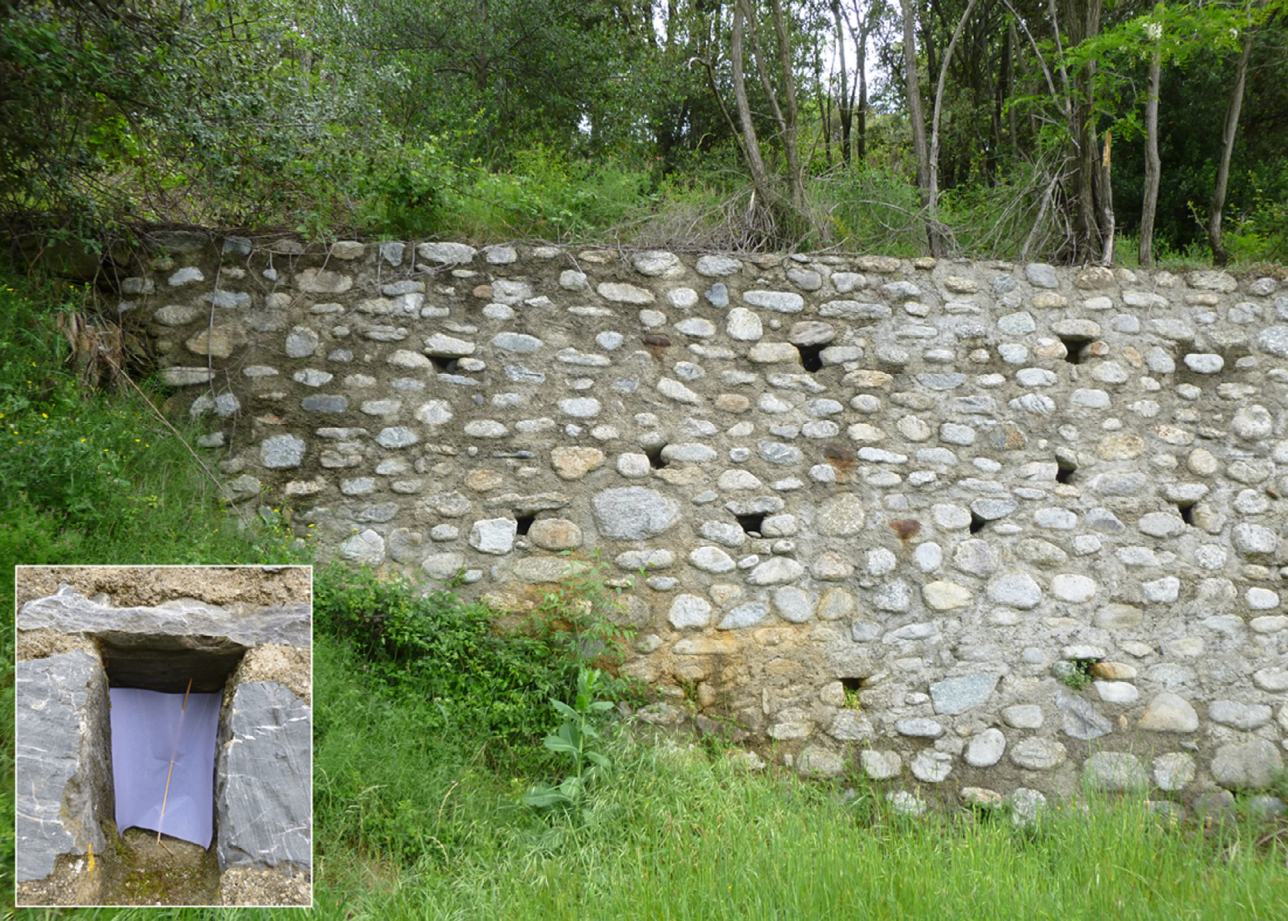
Station No. 3. Trapping wall with weep holes (or so-called “*barbacanes*” in French). Inset: a weep hole containing a sticky trap. Forefront: *Brachypodium phoenicoides* meadow along the edge of a road. Background: *Robinia pseudacacia* and *Quercus ilex.*

In the study area (mean elevation 200 m), 12 sampling stations located 2–3 km apart were selected using a purposive sampling method [12]. All stations except No. 1, 8 and 10 were located in wooded rural or periurban areas. Each had a retaining wall with drainage cavities (weep holes). Sandfly samples were obtained using flight interception traps (20 × 20 cm sticky paper that were stretched vertically in the weep holes) (Figure 3). From 3 June to 12 November 1981, the 12 stations were sampled on a 10-day basis from March to November (total number of traps: 4,263; total area: 341.04 m^2^; mean number of traps per station: 355.25; mean area per station: 28.42 m^2^; mean number of traps per 10-day period: 284). Sampled sandflies were placed in a 90° alcohol solution and identified. For each species, imago densities were calculated on the basis of the number of sandflies (P♂ + ♀) counted per m^2^ of sticky paper [2, 23, 27, 32]. The statistical analysis was focused on *P. ariasi* and *P. perniciosus,* the only confirmed vectors sampled at the site, using Kolmogorof-Smirnov, Wilcoxon and Friedman, Cochran and McNemar tests (SYSTAT^®^9 software) [33, 35].

### List of sampling stations and coordinates

Station No. 1 – Le Vila-locality. GPS coordinates (Coord.): latitude North (N) 42° 29,912′, Longitude east (E) 02° 42,651′. Elevation (Elev.): 150 m. Wall exposure (exp.): east southeast. Forest vegetation (For. veg.): *Quercus ilex* (*Qi*), *Q. suber* (*Qs*). Number of sampled traps (Tot. traps): 489.

Station No. 2 – Road (R) D15. Coord.: N 42° 29,901′, E 02° 42,528′. Elev.: 140 m. Exp.: E. For. veg.: *Qi*, *Qs*; Tot. traps: 73.

Station No. 3 – R D618 (Le Vila at Palalda). Coord.: N 42° 29,441′, E 02° 41,020′. Elev.: 280 m. Exp.: S. For. veg.: *Qi*, *Qs*. Tot. traps: 386. (Figure 3).

Station No. 4 – R D618 (Palalda 2 km). Coord.: N 42° 29,290′, E 02° 40,622′. Elev.: 260 m. Exp.: S. For. veg.: *Qi*. Tot. traps: 254.

Station No. 5 – Palalda-locality. Coord.: N 42° 29,087′, E 02° 40,437′. Elev.: 220 m. Exp.: SSE. For. veg.: *Qi*. Tot. traps: 332.

Station No. 6 – Palalda-locality. Coord.: N 420 29,067′, E 02° 40,315′. Elev.: 200 m. Exp.: SE*.* Tot. traps: 678.

Station No. 7 – R D618 (Palalda-Amélie). Coord.: N 42° 28,920′, E 02 40,203′. Elev.: 180 m. Exp.: SE. For. veg.: *Qi*, *Q. pubescens* (*Qp*). Tot. traps: 543.

Station No. 8 – Arles-localité. Coord.: N 42° 27,746′, E 02° 38,294′. Elev.: 200 m. Exp.: SE. Tot. traps: 712.

Station No. 9 – R F13 (Céret-locality). Coord.: N 42° 29,322′, E 02° 44,705′. Elev.: 120 m. Exp.: NE. Tot. traps: 203.

Station No. 10 – Céret-locality. Coord.: N 42° 29,430′, E 02° 44,800′. Elev.: 120 m. Exp.: E. Tot. traps: 305.

Station No. 11 – R D63 (Le Vila-Taillet). Coord.: N 42° 30,194′, E 02° 42,487′. Elev.: 235 m. Exp.: S. For. veg.: *Qs*, *Qp*, *Qi*. Tot. traps: 144.

Station No. 12 – R D63 (Le Vila-Taillet). Coord.: N 42° 30,182′, E 02° 42′,506′. Elev.: 240 m. Exp.: S. For. veg.: *Qs*, *Qi*, *Qp*. Tot. traps: 144.

### Corine biotope habitats

A 2,000 m^2^ area was delineated around each sampling wall to integrate the different natural or manmade habitats that could have an impact on sandfly abundance. These habitats (including walls and access roads) were classified according to Corine Biotope codes [1, 36]. Most of them corresponded to synsystematic units (phytosociological classification of J. Braun-Blanquet). For each station, the cover of each habitat was expressed as a percentage occupation within the delineated areas. The weep-hole retaining walls and access roads accounted for 18% of the overall area.

Habitat/station/sandfly relationships were described by normalized principal component analysis (PCA) [7, 34] on a matrix designed to correlate 12 sampling station records with 19 Corinne Biotope habitats (Table 1, Figures 9 and 10). In order to determine potential links between vegetation complexes and vector productivity, total sandfly densities were projected on the first PCA axis which pooled all of the main floristic characteristics in the records (ADE^®^4 software).

**Table 1.** Frequency (% cover per 2,000 m^2^) of Corine Biotope habitats in the 12 sampling stations (including trapping walls and access roads). These stations were highly modified by human activities, but holm oaks were still well represented in most of them (20–40%).

Corine Biotope codes	Corine Biotope habitats	St.	1	2	3	4	5	6	7	8	9	10	11	12	Average (%)
		Elev.	150 m	140 m	280 m	260 m	220 m	200 m	180 m	200 m	120 m	120 m	235 m	240 m	
		Exp.	S.E.	E.	S.	S.	S.E.	S.E.	S.E.	S.E.	N.E.	E.	S.	S.	
86.43	Open areas		15%	15%	20%	30%	15%	15%	15%	20%	25%	20%	15%	15%	18%
86	Towns, villages		30	30	•	15	50	25	•	•	60	60	•	•	18
45.3	*Quercion ilicis*		20	20	35	•	10	40	30	•	•	•	20	40	26
85.3	Gardens		•	20	•	•	•	•	•	15	15	20	•	•	6
41.85	*Celtis australis*		•	15	•	15	•	20	•	•	•	•	•	•	4
84.1	Tree rows		•	•	3	•	10	•	15	•	•	•	•	•	4
87.2	Ruderal communities		•	•	15	•	•	•	•	15	•	•	•	10	3
83.15	Fruit orchards		20	•	•	25	•	•	•	•	•	•	•	•	4
83.324	*Robinia pseudacacia*		10	•	15	•	•	•	•	30	•	•	•	•	3
32.21C	*Osyris* brush		•	•	•	•	5	•	5	•	•	•	•	•	0.8
45.216	*Quercus suber*		•	•	7	•	•	•	•	•	•	•	5	35	4
83.211	Traditional vineyards		•	•	•	15	•	•	•	•	•	•	•	•	1
83.3113	*European cypress*		•	•	•	•	10	•	•	•	•	•	•	•	0.8
62.2	Vegetated siliceous cliffs		•	•	•	•	•	•	35	•	•	•	•	•	2
44.5	*Osmundo-Alnion*		•	•	•	•	•	•	•	15	•	•	•	•	1
31.831	Bramble thickets		•	•	•	•	•	•	•	5	•	•	•	•	0.4
83.3112	Native pine plantations		•	•	•	•	•	•	•	•	•	•	30	•	2.5
41.714	*Quercus pubescens* forest		•	•	5	•	•	•	•	•	•	•	30	•	2.5
32.A	*Spartium junceum*		5	•	•	•	•	•	•	•	•	•	•	•	0.4

## Results and Discussion

1. At the end of the survey, 8,280 sandflies (P) were caught in a total of 4,263 traps (Table 2). When calculated in terms of m^2^ of sticky paper (both sides), the annual cumulative frequency of *P. ariasi*, *P. perniciosus* and *Sergentomyia minuta* was 24.27 P/m^2^. *S. minuta* was more abundant (5,600 specimens: 16.42 P/m^2^), followed by *P. ariasi* (2,297 specimens: 6.73 P/m^2^), and *P. perniciosus* (383 specimens: 1.12 P/m^2^). Stations with the highest abundance of *P. ariasi* (>8 P/m^2^, No. 3, 4, 11, 12) were south-facing. For *S. minuta,* stations 4 and 11 also had the highest abundances (44.83 P/m^2^ and 257.37 P/m^2^, respectively).

**Table 2.** Annual frequencies of the three sandfly species *(Phlebotomus ariasi*, *P. perniciosus*, *Sergentomyia minuta)* caught at the 12 selected sampling stations (“rational choice” method). There were considerable differences in sampled sandfly abundances between these stations (1.36–268.57 P/m^2^).

Stations	1	2	3	4	5	6	7	8	9	10	11	12	Total
Number of traps	489	73	386	254	332	678	543	712	203	305	144	144	4263
Surface area (m^2^)	39.12	5.84	30.88	20.32	26.56	54.24	43.44	56.96	16.24	24.4	11.52	11.52	341.04
*P. ariasi*	47	27	382	1049	44	60	53	16	46	11	103	459	2297
P/m^2^	1.2	4.62	12.37	51.62	1.65	1.1	1.22	0.28	2.83	0.45	8.94	39.84	6.73
*P. perniciosus*	38	8	32	188	10	25	8	5	19	12	26	12	383
P/m^2^	0.97	1.36	1.03	9.25	0.37	0.46	0.18	0.08	1.16	0.49	2.25	1.04	1.12
*S. minuta*	408	125	280	911	556	7	16	57	89	84	2965	102	5600
P/m^2^	10.42	21.4	9.06	44.83	20.93	0.12	0.36	1.00	5.48	3.44	257.37	8.85	16.42
P. total	493	160	694	2148	610	92	77	78	154	107	3094	573	8280
P/m^2^	12.6	27.39	22.47	105.70	22.96	1.96	1.77	1.36	9.48	4.38	268.57	49.73	24.27
2. The cumulative monthly total for the 12 stations was used to plot annual activity curves for both vectors. At Vallespir, *P. ariasi* thus had a bimodal distribution pattern, with the second peak in early autumn, whereas this species had a unimodal distribution in the Cévennes region (Figure 4). This pattern was likely due to bioclimatic differences, as indicated by the presence of cork oak, since the Pyrénées-Orientales stations had much milder climatic conditions than the southern Cévennes. These differences could be explained by the fact that *P. ariasi* thrives in humid-subhumid climatic conditions [23]. A similar phenomenon was observed with *P. perniciosus*, which is the most thermophilic sandfly species. At Vallespir, as in the Cévennes, this species had a unimodal distribution, while it has a bimodal pattern in the vicinity of Tunis, where semiarid climatic conditions prevail (Figure 5) [3]. However, there could have been a temporal shift in the maximum activity peaks between stations. At station No. 12, the peak for *P. ariasi* was thus around 20 June, whereas it occurred around 20 July at station No. 4 (Figure 6). Excluding the fact that the corresponding walls had identical exposure, this shift could have been due to other factors, such as the extent of solar radiation or the presence of vertebrate hosts.

**Figure 4.**
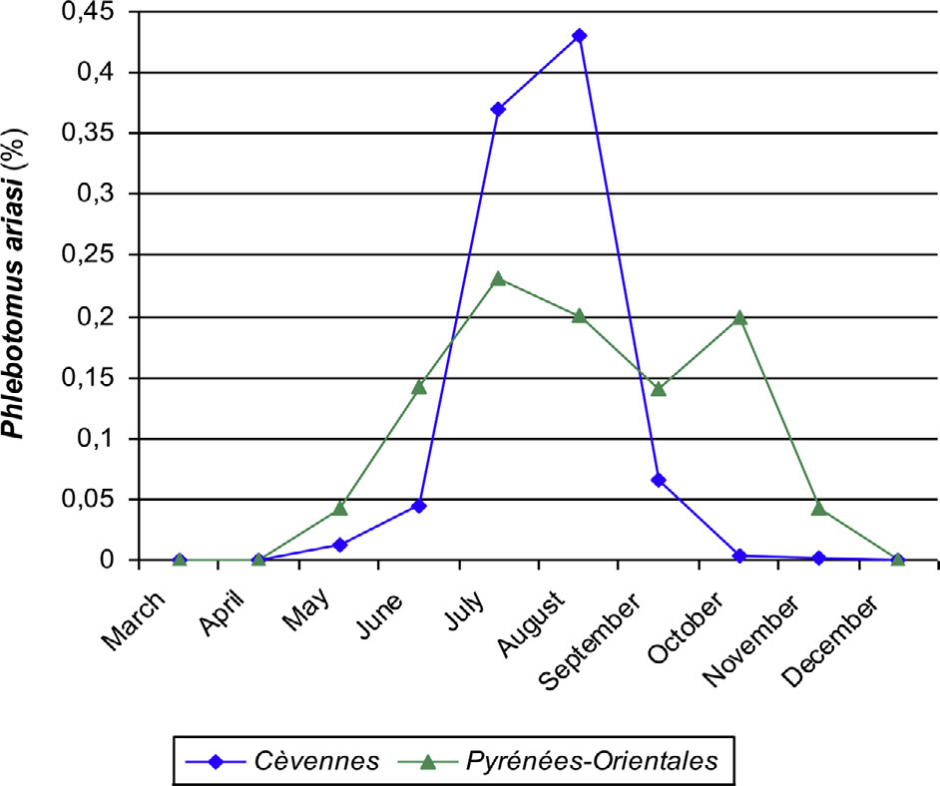
Monthly frequency distribution of *Phlebotomus ariasi* ♂ + ♀ (sampling with sticky-paper traps): in the Cévennes region in 1960 and in Pyrénées-Orientales region in 1981. The subhumid bioclimatic conditions in the Pyrénées-Orientales could explain the bimodal distribution pattern and the autumn frequency spread.

**Figure 5.**
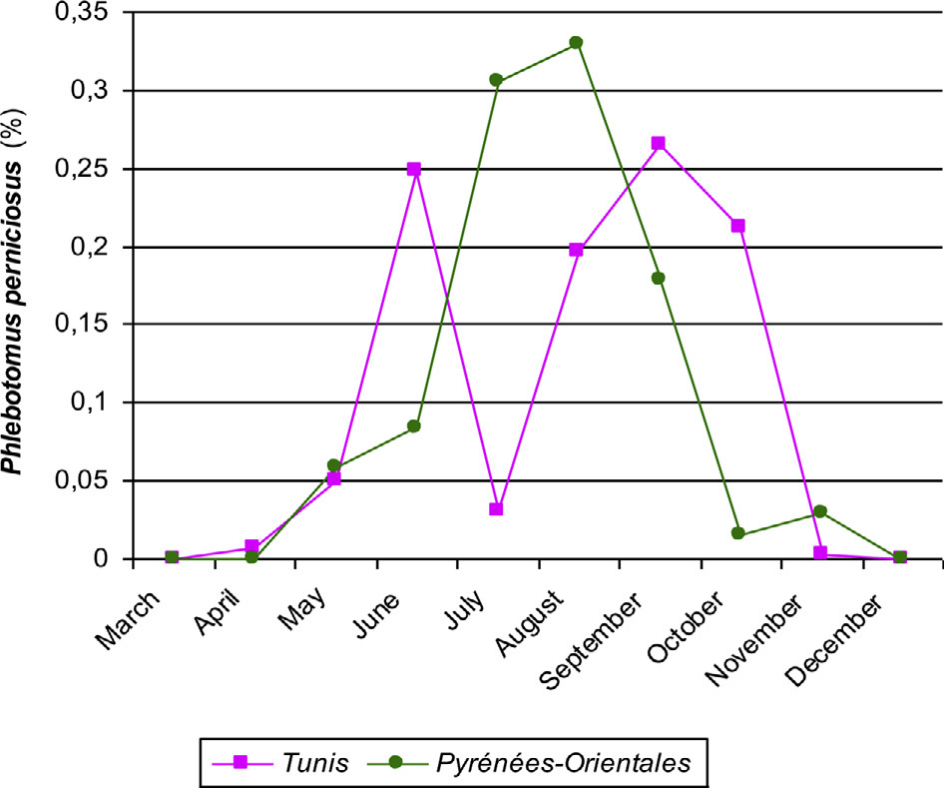
Monthly frequency dynamics of *Phlebotomus perniciosus* in Pyrénées-Orientales region (1981) and in Tunisia (1960). In the vicinity of Tunis, under a semiarid Mediterranean bioclimate, these frequencies had a biphasic distribution pattern (summer diapause?). At Vallespir, in subhumid bioclimatic conditions, a unimodal distribution was noted.

**Figure 6.**
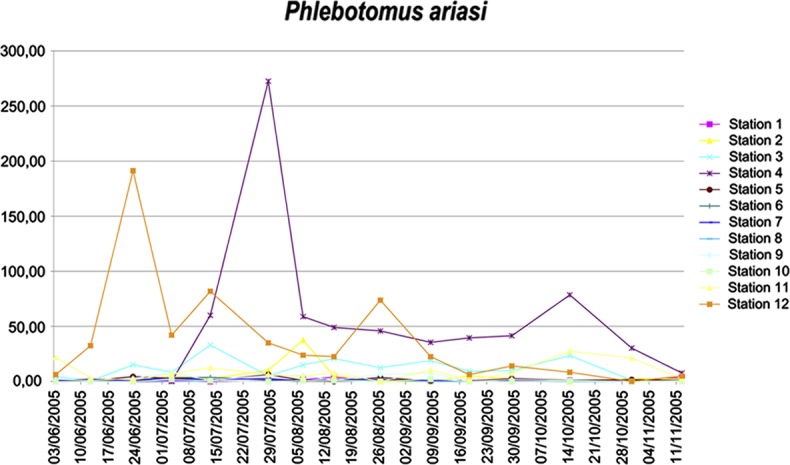
10-day frequency distribution for *Phlebotomus ariasi* at the 12 sampling stations. The stations with the highest abundance (No. 4 and No. 12) showed a marked shift in maximum peak abundance: in July-June for No. 14 and July for No. 4. There was a similar shift in the lower second peak. This shift was likely related to the characteristics of the two trapping walls.
3. *P. ariasi* was clearly the most abundant of the two vectors present at the site: for 4,263 sticky paper traps (surface area 341.04 m^2^), a total of 2,297 specimens (6.73 P/m^2^) were sampled, as compared to only 383 for *P. perniciosus* (1.12 P/m^2^). Moreover, there were substantial between-station differences in densities of both species. At stations No. 4 and No. 12, for *P. ariasi*, a total of 51.62 P/m^2^ and 39.84 P/m^2^, respectively, were sampled, whereas at the 10 other sites, the frequencies never exceeded 13 P/m^2^. For *P. perniciosus*, the two stations with the greatest densities (No. 4, No. 11) had a total of 9.25 P/m^2^ and 2.25 P/m^2^, respectively, whereas the frequencies at the remaining stations ranged from 0.08 P/m^2^ (No. 8) and 1.36 P/m^2^ (No. 2) (Table 2).
4. The statistical analyses confirmed these findings. The comparison of vector densities revealed significant differences between the 12 stations, i.e. each had a specific sandfly abundance pattern with respect to both *P. ariasi* (Figure 7) and *P. perniciosus*.

**Figure 7.**
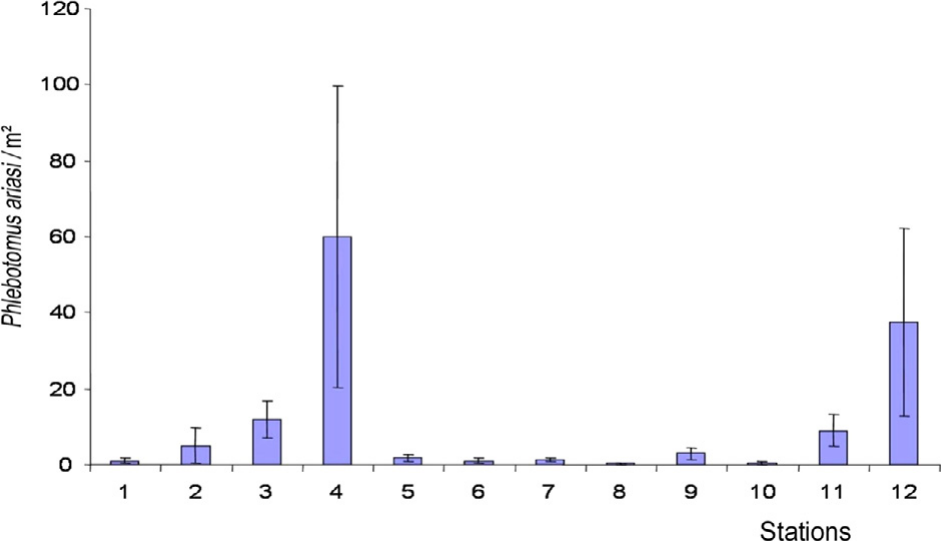
*Phlebotomus ariasi*: mean annual density per station (P ♂ + ♀ / m^2^). These frequencies did not have a normal distribution (Kolmogorov-Smirnov test, *p* < 0.01). The Friedman test, *p* < 0.001 (nonparametric, matched by date), showed that the frequencies were significantly different between the 12 stations, i.e. each station had its own specific sandfly density.
5. Moreover, although the sandfly densities were specific to each station, this was not the case for their variations. Between samplings (10-day sampling periods), the variation trends were not significantly different: the curves were parallel and in the same direction for the 12 stations (Figure 8).

**Figure 8.**
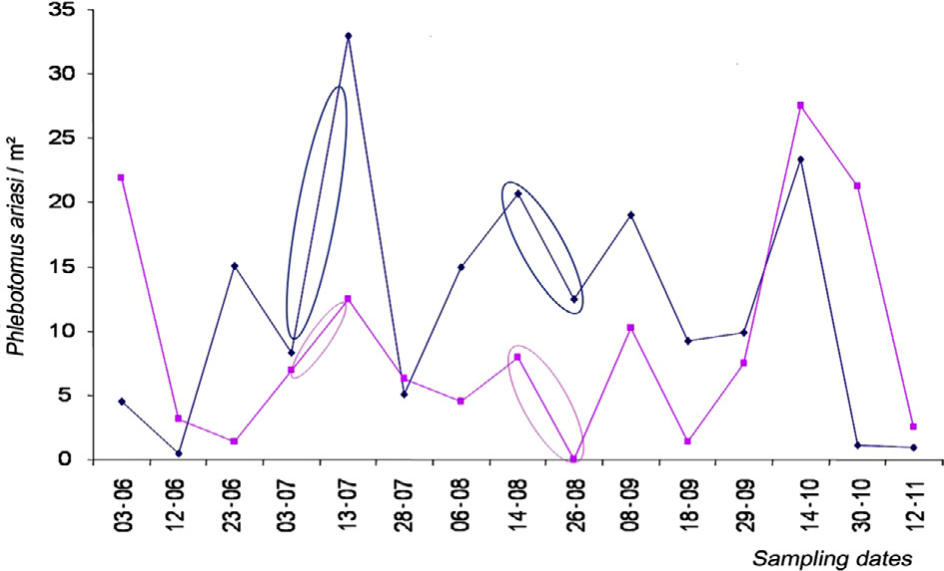
10-day variations in *Phlebotomus ariasi* densities-sampling results for stations No. 3 (red) and No. 11 (blue). The 10-day variation patterns did not significantly differ between stations (Cochran’s Q test, *p* = 0.949, nonparametric, matched by date).
6. Projection of the vector abundances on the first PCA axis did not reveal any relationship between the sampled sandfly abundance of the trapping walls and their phytological environment, expressed in terms of the cover of the different Corine Biotope habitats (Figures 9, 10).

**Figure 9.**
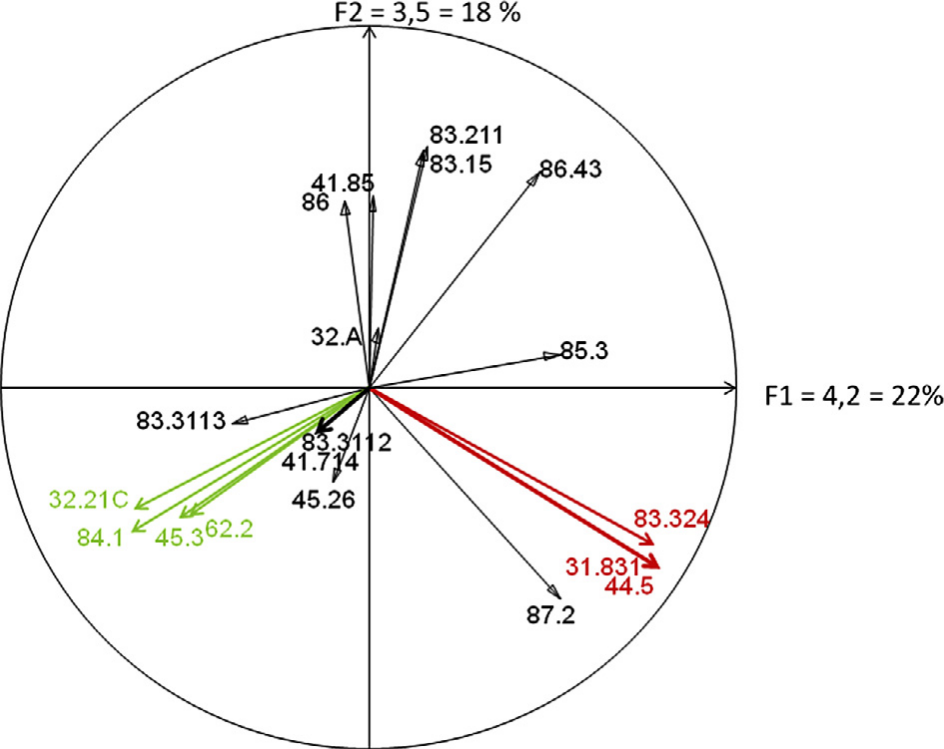
Circle of correlation from a normalized PCA based on the data in Table 1 (19 Corine Biotope codes × 12 sampling station records). These correlations led to the identification of the Corine Biotope habitats most frequently associated in each of the 12 records. The F1 axis thus compares records containing wild *Robinia* stands (83.324), western Mediterranean riparian forests (44.5) and brambles (31.831) with those containing *Osyris alba* brush (32.216), tree rows (84.1), and holm oak thickets (45.3) and vegetation cover on siliceous rocks (62.2). The *F*2 axis compares records that associate orchards (83.15) and vineyards (83) with those that do not. The method used to differentiate the stations according to their phytoecological profile … and nothing else!

**Figure 10.**
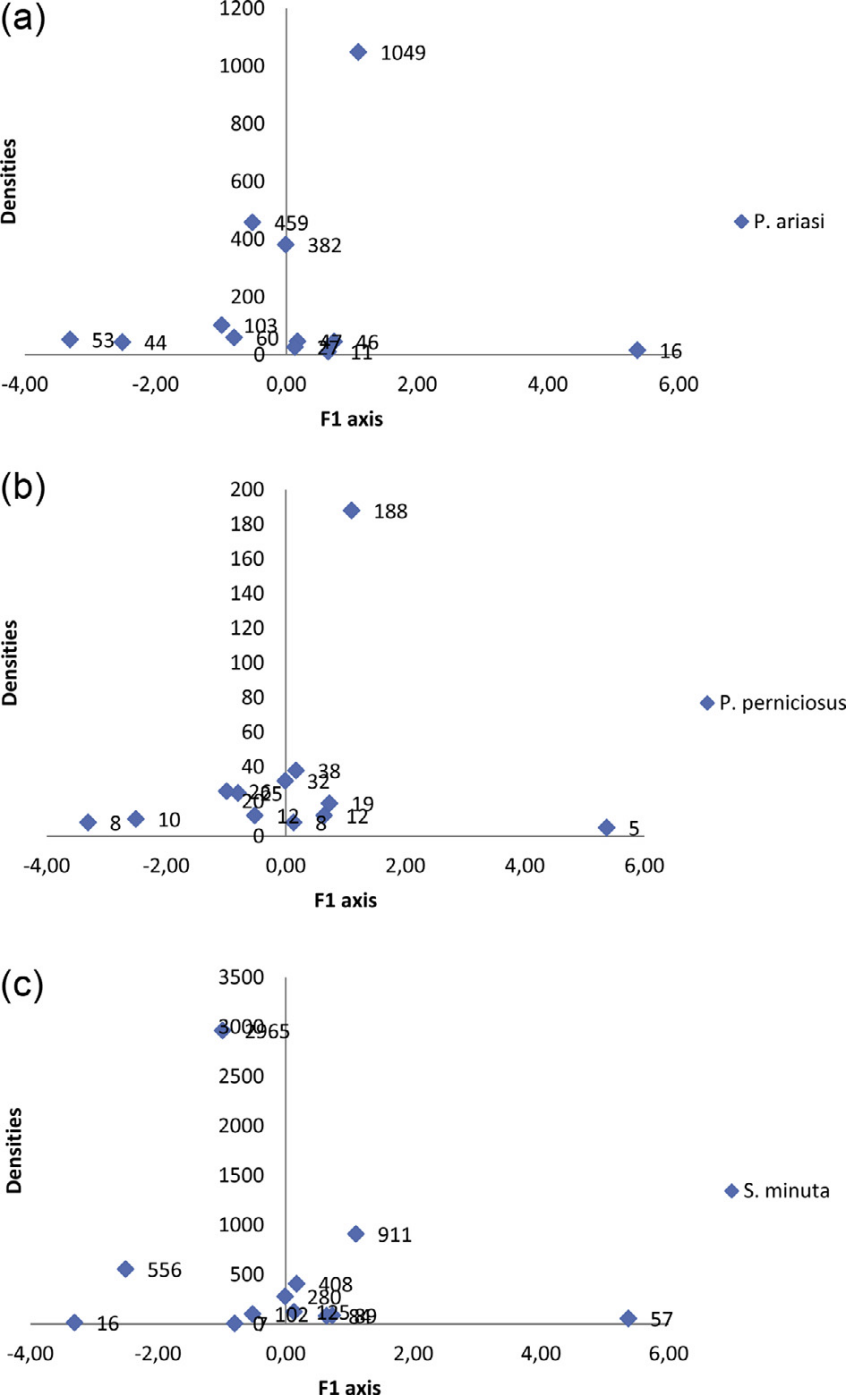
Projection of *Phlebotomus ariasi* (a), *P. perniciosus* (b) and *Sergentomyia minuta* (c) densities at sampling stations on the normalized PCA F1 axis. Among the readings grouped at the origin on the F1 axis, there are two habitat groups that are opposed in Figure 9 (circle of correlations). There is therefore no gradual relationship between the vector abundance and the environmental (habitat) trends. Corine Biotope habitats thus do not seem to be suitable for drawing up a vector sampling plan.

## Conclusion and prospects

The present results led to the following conclusions:

- At the study site, the per-station *P. ariasi* abundances were significantly higher than those of *P. perniciosus.* However, this species was not as scarce as it was in the southern Cévennes region. This difference could be explained by the bioclimatic conditions, i.e. humid Mediterranean climate in the Cévennes and subhumid at Vallespir. These climatic differences could also explain the *P. ariasi* variation patterns, i.e. monophasic in the Cévennes and diphasic at Vallespir.
- The significant between-station differences in sampled sandfly abundance were due to the specific environmental conditions at each station (geopedology, orientation and shadows cast on the trapping wall, animal occupation of the weep holes, nearby livestock) [18, 28]. Note that the weep holes served as roosting sites, not as breeding sites
- During many surveys, our attempts to isolate larvae or nymphs in weep-hole soil were always fruitless. However, the constant high abundance sampled in some especially attractive walls could be explained by the high movement capacity of imagos [30].
- The parallelism in the 10-day variations could have been due to a single factor, i.e. likely weather related (storm, heat wave, etc.), taking place accidentally and sporadically at all stations.
- The multivariate analysis did not reveal any association between sandfly frequencies and the phytoecological environment of the sampling stations (Figures 9 and 10). As we noted, these associations occurred with certain physical or biotic properties specific to each wall. More generally, the cartographic typology of the area according to the Corine Biotope system or its derivatives (Corine Land Cover, EUNIS [15, 17]) cannot be recommended as a vector abundance indicator, especially in the definition of zero points. By their phytosociological nature, the corresponding maps should be used for studies on landscape modifications resulting from climate change, fire, flooding, cropping or non-native plant invasions. However, these maps have a real value in applied research, e.g. in forest management (see French Forestry Office). Finally, they can still be used as density indicators to characterize certain zoological groups on the condition that they are closely tailored to specific botanical taxa or syntaxa. This category includes strict pests, specific pollinators, specialized herbivores and animals with a narrow ecological niche. In other cases, caution is necessary [21], especially since Corine Biotope maps are not the only way to express vegetation-indicator relationships. This was the case in studies carried out in Morocco to identify the preferred bioclimatic conditions of sandflies. In this country, the only phytoclimatic map, drawn up on the basis of vegetation layers (from humid to arid), made it possible to attribute a bioclimatic value to each inventoried sandfly species, and especially to specify the current or future geographical distribution (global warming) of *Leishmania* spp. vectors [23]. Otherwise, in metropolitan France, a very successful phytoecological map was drawn up of biotopes for pre-adults of *Aedes* spp. (Diptera-Culicidae). The detection of eggs in the litter of halophyte plants revealed a close relationship between some plant species (*Salicornia* spp., *Scirpus maritimus)* and certain Aedinae species (*Aedes caspius, A. detritus*). This relationship was dependent on the egg-laying behaviour of gravid females, which were found to often only lay eggs on one or two species of plants growing along narrow strips in lagoon environments*.* The focus has thus readily shifted from “egg laying sites” to the phytoecological mapping of “breeding sites”, which has turned out to be a remarkable operational tool that has been very successful in the control of pest mosquitoes in Mediterranean coastal regions [10, 24, 25].
- Finally, the current results should be considered as a starting point for further research. At Vallespir, after more than 30 years, the same protocol should be applied at the same sampling stations. This type of operation was recently undertaken in the lower Cévennes region [19, 31], which generated promising results. At Vallespir, if the sandfly density modifications and geographical distributions noted here are confirmed, it would be of interest to supplement this work by more in-depth taxonomic and genetic analyses, both in terms of specific taxa (*P. sergenti* is already present in Pyrénées-Orientales region [29]), and within the same species (modification in population-based polymorphism, selection of thermophilic variants, drift [30], etc.). Future epidemiological research focused on the impact of climate change or of agronomic-silvicultural modifications should be very cautiously carried out, especially with respect to vector sampling and the use of phytoclimatic maps as vector density indicators.
